# Isochromosome Yp and jumping translocation of Yq resulting in five cell lines in an infertile male: a case report and review of the literature

**DOI:** 10.1186/1755-8166-6-36

**Published:** 2013-09-10

**Authors:** Morteza Hemmat, Omid Hemmat, Fatih Z Boyar

**Affiliations:** 1 Cytogenetics Department, Quest Diagnostics Nichols Institute, 33608 Ortega Highway, San Juan Capistrano, CA 92690, USA; 2 Ostrow School of Dentistry, University of Southern California, Los Angeles, CA, USA

**Keywords:** Isochromosome Yp, Jumping translocation Yq

## Abstract

**Background:**

Jumping translocations are a rare type of mosaicism in which the same portion of one donor chromosome is translocated to several recipient chromosomes. Constitutional forms of jumping translocations are rare, and the 48 cases reported to date have been associated with both normal and abnormal phenotypes. Concurrence of isochromosome (i) of one arm and translocation of the other is also rare, with seven reported cases. We describe a unique case involving concurrence of i(Yp) and a jumping translocation of Yq to the telomere of chromosomes 12q and 17q, which resulted in five cell lines.

**Case presentation:**

The patient, an otherwise healthy 35-year-old man, was referred for cytogenetic studies because of absolute azoospermia. He had elevated levels of follicle stimulating hormone and luteinizing hormone, consistent with abnormal spermatogenesis, and decreased levels of free testosterone and inhibin B. G-banded chromosome analysis revealed a mosaic male karyotype involving five abnormal cell lines. One of the cell lines showed loss of chromosome Y and presence of i(Yp) as the sole abnormality. Three cell lines exhibited jumping translocation: two involved 17qter, and the other involved 12qter as the recipient and Yq as the common donor chromosome. One of the cell lines with der(17) additionally showed i(Yp). The other der(17) and der(12) cell lines had a missing Y chromosome. All five cell lines were confirmed by FISH. Subtelomric FISH study demonstrated no loss of chromosome material from the recipient chromosomes at the translocation junctions.

**Conclusions:**

We postulate that a postzygotic pericentromeric break of the Y chromosome led to formation of isochromosome Yp, whereas Yq formed a jumping translocation through recombination between its internal telomere repeats and telomeric repeats of recipient chromosomes. This in turn led to either pairing or an exchange at the complimentary sequences. Such translocation junctions appear to be unstable and to result in a jumping translocation. Cryptic deletion or disruption of AZF (azoospermic factor) genes at Yq11 during translocation or defective pairing of X and Y chromosomes during meiosis, with abnormal sex vesicle formation and consequent spermatogenetic arrest, might be the main cause of the azoospermia in our patient.

## Background

Jumping translocations, a term first introduced by Lejeun and colleagues [1], are a rare type of mosaicism involving the same portion of one donor chromosome translocated to several different recipient chromosomes. They usually occur somatically, in both constitutional and acquired chromosomal abnormalities, and occur in various pathologic conditions. Constitutional forms of jumping translocations are very rare and have varying clinical impact; the 48 cases reported to date have been associated with both normal and abnormal phenotypes [1-11]. Acquired jumping translocations, on the other hand, have been more commonly observed in hematologic malignancies [12].

A jumping translocation probably arises through multiple events of somatic recombination in early development [7,13]. Regions of repetitive DNA sequences such as centromeres, pericentromeres and telomeres, have been implicated to play a critical role in the events leading to the formation of jumping translocations. Telomere-like repeats, also called interstitial or internal telomeres [13,14], are generally present in pericentric regions and represent nonfunctional chromosomal elements [15]. These internal telomere repeats have been proposed to be hotspots for recombination [13,16]. Such hotspots have also been confirmed by evidence pointing toward preferential involvement of the heterochromatin region of donor chromosomes [17] and telomere ends of recipient chromosomes [14]. Additionally, heterochromatic regions may undergo decondensation during early development, leading to increased recombination at the pericentrometric regions of some of the chromosome arms [17]. However, these translocation junctions appear to be unstable and to result in jumping translocations [5]. The fact that not all such rearrangements result in jumping translocations [15,18,19] suggests that sequences from both chromosomes at the junction influence the stability of the fusion.

Seven instances of concurrent isochromosome of the short arm, i(Yp), and translocation of the long arm of a single chromosome onto a telomere of a nonhomologous chromosome have been described [20-26]. Concolino and colleagues suggested that alpha-satellite fission of the chromosome can result in an isochromosome of the short arm and deletion of the long arm [27]. Moreover, Petit noted that chromosomal fission within the alpha satellite is likely to form a stable deletion, of one chromosomal arm, whereas fragments with breaks at the junction of alpha and beta sequences may translocate to the telomere of another chromosome [9]. However, one function of telomeres is to prevent chromosomes from fusing with other chromosomal fragments [28]. In several organisms in which telomere shortening is seen, telomeres lose the ability to protect against chromosome fusion well before all telomere sequence is lost. Perhaps a minimum length is needed to form a *t*-loop; once they are sufficiently short, they can no longer protect the end [29]. Overall, this somatic recombination provides a protective mechanism through which the fragment can be maintained within a cell [30].

Azoospermia, when no sperm can be detected on two separate semen samples, is found in up to 20% of infertile men [30]. Genital tract obstruction and defective spermatogenesis are the principal causes of azoospermia [31]. Chromosomal abnormalities are present in 7% of infertile men and in 10% to 15% of azoospermic men [32,33]. Among the karyotypic abnormalities found in azoospermic men, sex chromosome abnormalities predominate [34,35]. Translocations involving the Y chromosome have been reported in association with male infertility and azoospermia [36]. In the most common form, the heterochromatic region of Yq is translocated. A chromosomal breakpoint at band Yq11 is rarely described. Moreover, isochromosome Yp, which results in duplication of the short arm and loss of the long arm, has been reported in association with azoospermia. Two mechanisms have been proposed for male infertility. First, the AZF (azoospermic factor) gene, which is located at Yq11 and is critical for spermatogenesis, may be deleted secondary to a microdeletion, rearrangement, or complete loss as a result of the translocation mechanism [36]. The second explanation is that a defective X–Y pairing during meiosis, with abnormal sex vesicle formation, could lead to spermatogenetic arrest [37-40].

We describe the first reported case involving a de novo isochromosome Yp and jumping translocation of Yq, which resulted in five cell lines and was associated with azoospermia in an infertile man.

## Case presentation

### Clinical report

The patient was an apparently healthy 35-year-old man with infertility and absolute azoospermia. His physical examination was normal except for slight testicular hypotrophy. No sperm were found in any of the three routine semen analyses, and the elevated serum concentrations of FSH (17 IU/L; normal range 3–7 IU/L) and LH (12 IU/L; normal range 3–8 IU/L) suggested abnormal spermatogenesis. The free testosterone (286 ng/dL; normal range 450–950 ng/dL) and inhibin B (26 pg/mL, normal range 80–270 pg/mL) levels were low. The patient was referred to our laboratory for chromosome analysis.

## Material and methods

### Cytogenetic and Fluorescence In Situ Hybridization (FISH) studies

The chromosomes of the patient and his parents were studied in peripheral blood mitoses, after culturing and G banding by standard techniques.

The results were confirmed by performing fluorescence *in situ* hybridization (FISH) with probes for the Yqh region (DYZ1), the sex determining region Y (SRY) gene, and the chromosome X centromere (DXZ1) probe (Vysis/Abbott, Inc., Downers Grove, IL). The translocation junctions of recipient chromosomes were illustrated by chromosome specific subtelomeric probes for chromosomes 12 and 17 (TelVysion; Vysis, Downers Grove, IL, USA). All FISH studies were performed according to the manufacturer’s protocol. Fluorescence images were captured with a Nikon epifluorescence microscope and analyzed by ISIS software (MetaSystems, Altlussheim, Germany).

## Results

**Figure 1:**
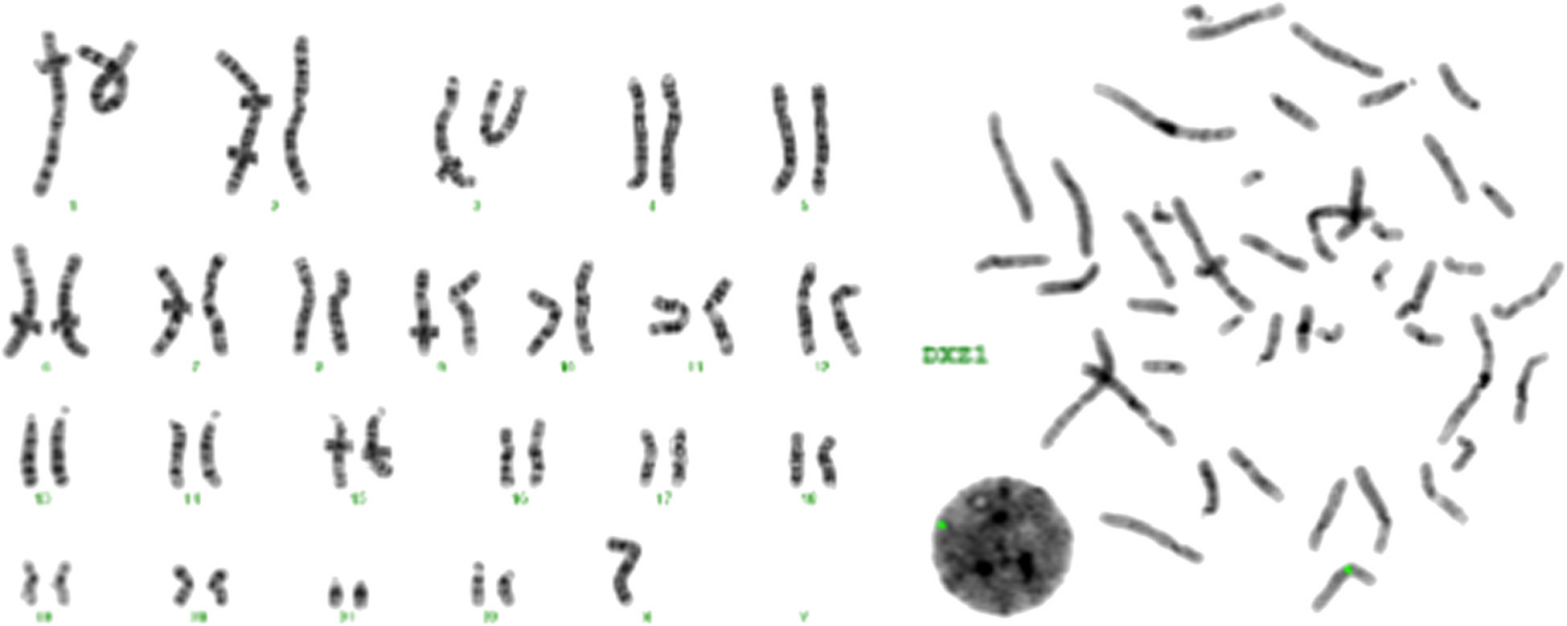
**A metaphase showing loss of chromosome Y.** The left side shows the G-banded karyotype and the right side is the inverted DAPI image of metaphase using Yqh (green), SRY gene (red), and X centromere (green) probes.

**Figure 2:**
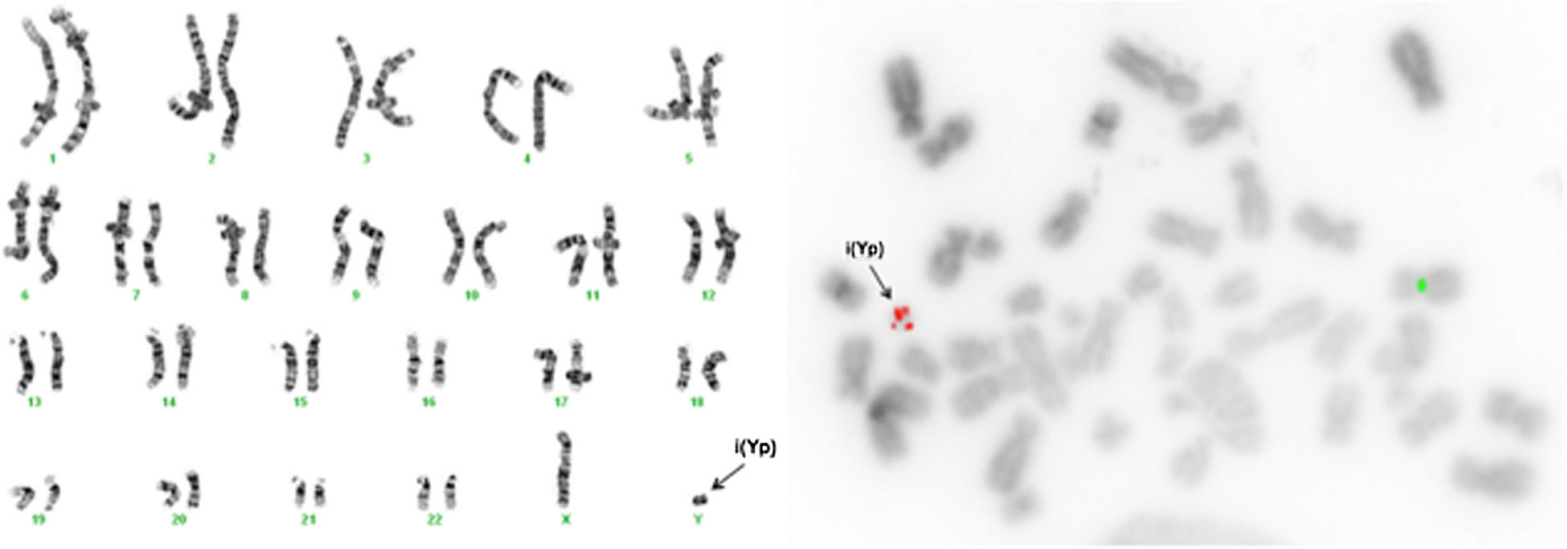
**A metaphase showing duplication of Yp resulting in isochromosome Yp and loss of Yq.** The left side shows the G-banded karyotype and the side image is the inverted DAPI image of metaphase using Yqh (green), SRY gene (red), and X centromere (green) probes.

**Figure 3:**
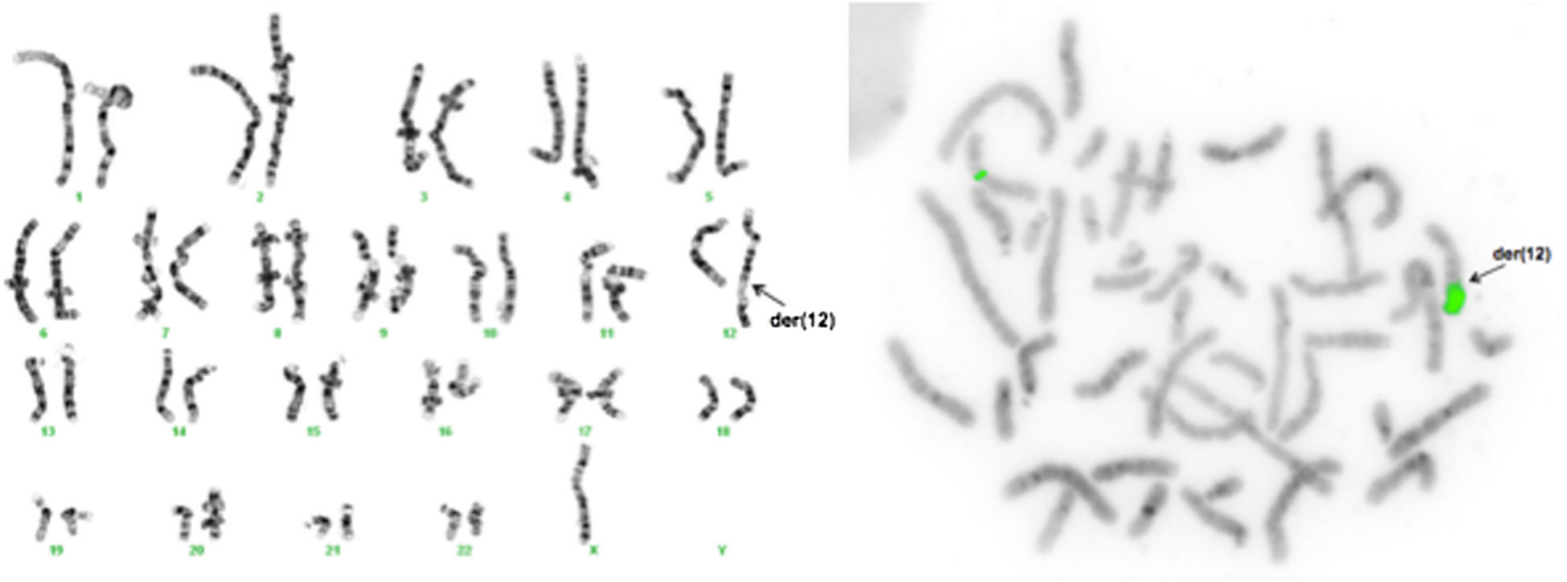
**A metaphase showing translocation of Yq to 12qter resulting in derivative chromosome 12 and loss of Y chromosome.** The left side shows the G-banded karyotype and the right side is the inverted DAPI image of metaphase using Yqh (green), SRY gene (red), and X centromere (green) probes.

**Figure 4:**
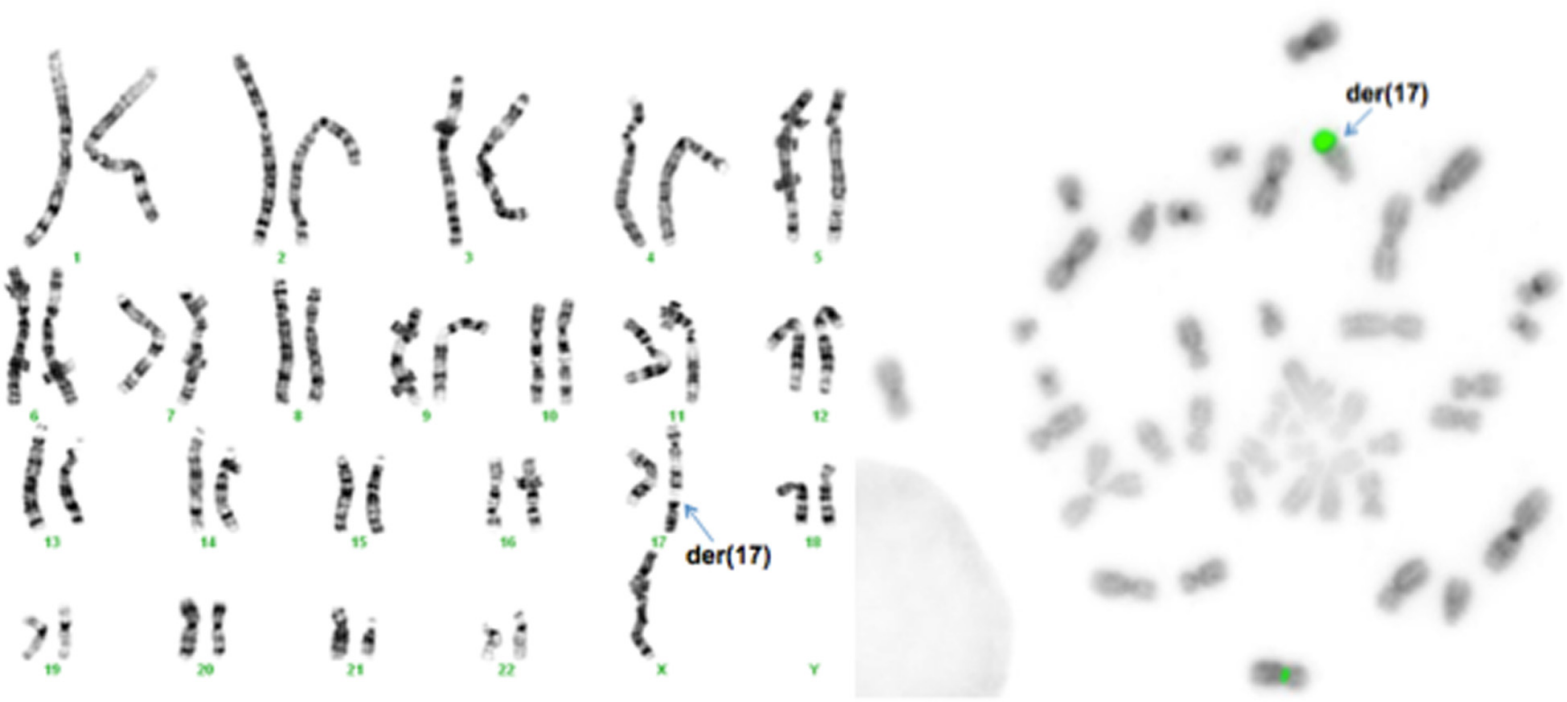
**A metaphase showing translocation of Yq to 17qter resulting in derivative chromosome 17 and loss of Y chromosome.** The left side shows the G-banded karyotype and the right side is the inverted DAPI image of metaphase using Yqh (green), SRY gene (red), and X centromere (green) probes.

**Figure 5:**
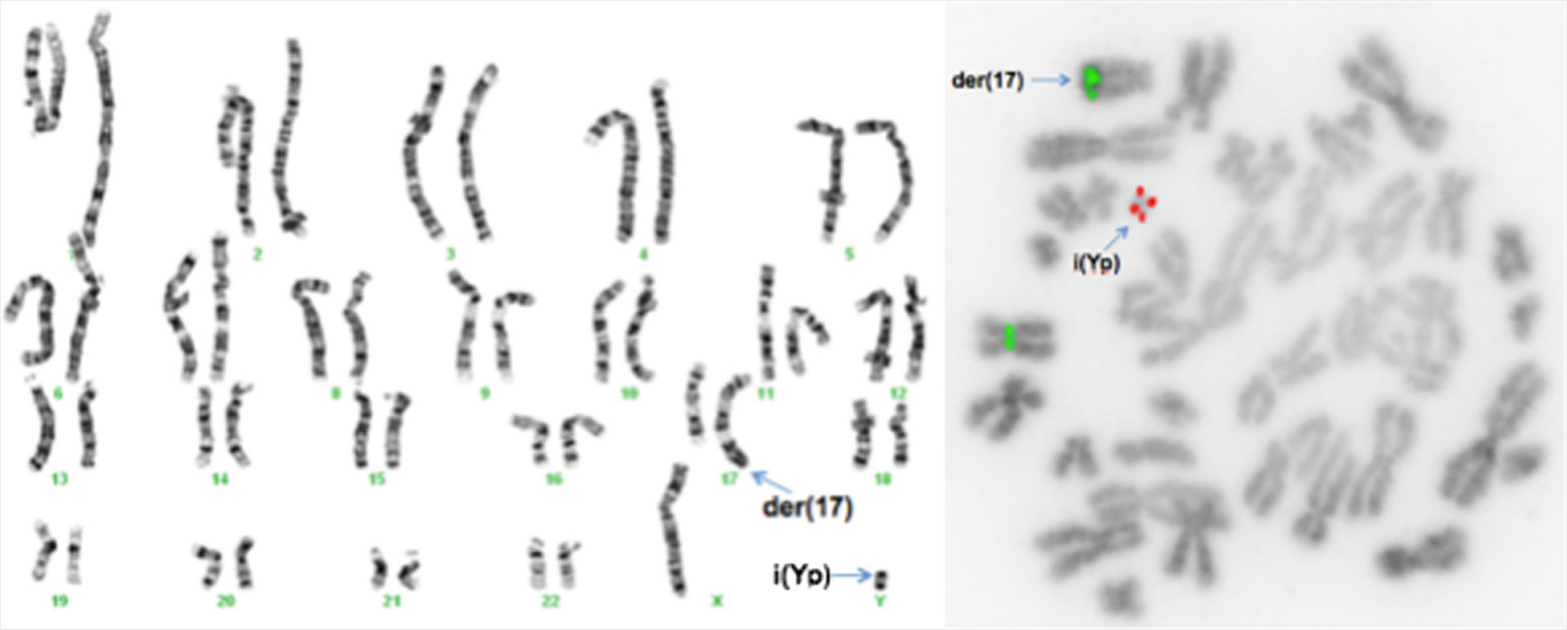
**A metaphase showing translocation of Yq to 17qter resulting in derivative chromosome 17 and duplication of Yp resulting in isochromosome Yp.** The left side shows the G-banded karyotype and the right side is the inverted DAPI image of metaphase using Yqh (green), SRY gene (red), and X centromere (green) probes.

The analysis of 50 metaphases and subsequent FISH studies revealed the presence of five cell lines in the proband, all with an abnormal chromosome Y. One cell line showed loss of chromosome Y (Figure 1; 6% of metaphases) and the other showed i(Yp) with loss of Yq (Figure 2; 6% of metaphases) as the sole abnormality. Two other cell lines represented derivative chromosome 12 (Figure 3; 8% of metaphases) or 17 (Figure 4; 28% of metaphases) due to fusion of Yq to the telomere of their long arms. The fifth cell line showed der(17) and i(Yp) as the second abnormality (Figure 5; 52% of metaphases). Additional count of 30 cells to the routinely 20 cells were in order to reduce the possibility of a 47,XXY cell line in peripheral blood. Both parents had normal G-banded karyotypes.

**Figure 6:**
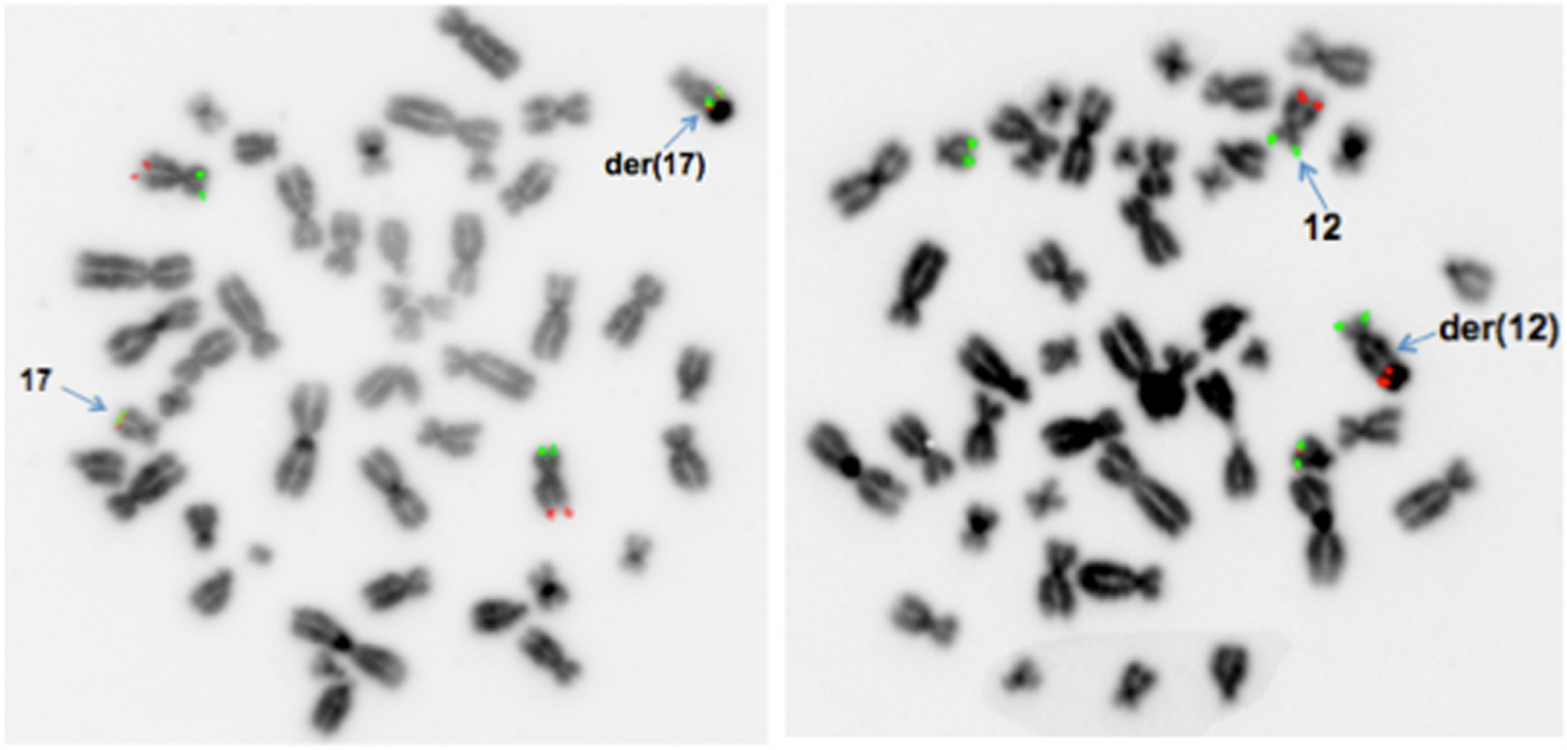
**Inverted Dapi image of metaphases using subtelomeric probes for the long arm (green) and short arm (red) of chromosome 12 (right image) and chromosome 17 (left image).** Subtelomeric probe signals (green) at the fusion junctions of both recipient chromosomes shows that there is no loss of chromosomal materials at these regions.

The hybridization of two SRY probes at i(Yp) in a mirror image configuration confirmed that this chromosome consisted of two short arms (Figures 2 and 5). The translocation of the long arm of Y into two different telomeric regions (17q and 12q) was confirmed using the Yqh probe (Figures 3, 4, and 5). Detection of the 12q and 17q subtelomeric probe signals confirmed that there was no loss of chromosomal materials at the translocation junctions (Figure 6).

Based on the results obtained from the chromosome and FISH analysis, the karyotype of our patient was designated as:

Mos46,X,idic(Y)(q11.1),der(17)t(Y;17)(q11.1;q25.3)[26]/45,X,der(17)t(Y;17)(q11.1;q25.3)[14]/45,X,der(12)t(Y;12)(q11.1;q24.33)[4]/46,X,i(Y)(q11.1)[3]/45,X[3].

## Conclusions

Constitutional jumping translocations are very rare, and only 7 cases to date have involved concurrent isochromosomes of one arm and translocations of the other [20-26]. The present case is unique, in that it exhibited concurrence of i(Yp) and jumping translocation of Yq as a result of fusion to the telomeres of chromosomes 12q and 17q.

Characteristics common to all eight reported cases (including the present case) of jumping chromosome with concurrent isochromosome and translocation of a single chromosome include a de novo origin, an initial centromeric or pericentromeric break, a monocentric appearance of both the isochromosome and the derivative, and absence of demonstrated chromatin loss. FISH study using subtelomeric probes demonstrated that both recipient chromosomes were intact, with no loss of chromosome material. Moreover, both parents had normal G-banded karyotypes, indicating that the chromosomal abnormalities in their son were de novo. Our case differed from the prior seven cases in having jumping translocation rather than a stable translocation.

These aberrations must have been initiated by a pericentromeric break of Y chromosome at Yq11.1, yielding two chromosomal fragments. The short arm carrying the centromere evolved into an isochromosome, and the acentric long arm fused to the telomeres of the recipient chromosomes. This is possibly the result of a natural mechanism that maintains both fragments throughout cell divisions. We postulate that the pericentromeric region of chromosome Y had a complementary sequence to the telomeric repeat of chromosomes 12 and 17, which led to either pairing or an exchange at the complimentary sequences. In either case, Reddy and colleagues [5] proposed that a single-stranded DNA or a double/triple-stranded DNA at the junction might cause the instability observed in jumping translocations.

Five cell lines evolved from the time of pericentromeric break until regaining complete stability by forming an isochromosome and recombining with the telomeres of two other chromosomes over the course of multiple cell divisions. One of the cell lines showed complete loss of chromosome Y; two cell lines maintained the translocation of Yq to 12q or 17q in the presence or absence of i(Yp); and one cell line showed i(Yp) and deletion of Yq. The events that led to stability probably occur only during early development [3], which resulted in a limited number of cell lines. The zygotic origin of isochromosomes and chromatin instability during early zygote division [30] lends support to this assumption. So, it can also be postulated that this is a de novo process and a post-fertilization event.

The azoospermia and resulting infertility in our patient might be as a result of 1) cryptic deletion or disruption of AZF genes at the Yq11.1 breakpoint (translocation’s junction); 2) defective pairing of X and Y chromosomes during meiosis, which could have led to abnormal sex vesicle development and consequent spermatogenetic arrest.

## Ethical approval and consent

These studies were performed on anonymized samples received in the clinical laboratory and thus were exempted from the requirement for consent by an opinion for the Western institutional review board.

## Competing interests

The authors declare that they have no competing interest.

## Authors’ contributions

The authors performed analysis, interpretation of the results, drafting and finalizing the manuscript. All authors read and approved the final manuscript.
